# Pediatric Cardiac Arrest Secondary to Guillain-Barré Syndrome-Induced Dysautonomia

**DOI:** 10.3390/children12101379

**Published:** 2025-10-13

**Authors:** Po-Jung Chen, Yi-Ting Cheng, Shao-Hsuan Hsia, Oi-Wa Chan, En-Pei Lee, Kuang-Lin Lin, Jainn-Jim Lin

**Affiliations:** 1 Department of Pediatric Cardiology, Chang Gung Children’s Hospital and Chang Gung Memorial Hospital, Chang Gung University College of Medicine, Taoyuan 33342, Taiwan; b9805015@cgmh.org.tw; 2 Division of Pediatric Neurology, Chang Gung Children’s Hospital and Chang Gung Memorial Hospital, Chang Gung University College of Medicine, Taoyuan 33342, Taiwan; b9902093@cgmh.org.tw (Y.-T.C.); lincgh@cgmh.org.tw (K.-L.L.); 3 Department of Physician Assistant, University of Maryland Eastern Shore, Princess Ann, MD 21853, USA; shsia@cloud.cgmh.org.tw; 4 Division of Pediatric Critical Care Medicine and Pediatric Neurocritical Care Center, Chang Gung Children’s Hospital and Chang Gung Memorial Hospital, Chang Gung University College of Medicine, Taoyuan 33342, Taiwan; ai3333@cgmh.org.tw (O.-W.C.); lnp1511@cgmh.org.tw (E.-P.L.); 5 Study Group for Intensive and Integrated Care of Pediatric Central Nervous System (iCNS Group), Chang Gung Children’s Hospital, Taoyuan 33342, Taiwan

**Keywords:** Guillain-Barré syndrome, children, dysautonomia, cardiac arrest, polyneuropathy, IVIG, plasmapheresis

## Abstract

**Highlights:**

**What are the main findings?**

- Pediatric Guillain-Barré Syndrome (GBS) can progress rapidly to life-threatening dysautonomia.
- Early cardiac arrest due to autonomic dysfunction may occur even in initially mild cases.

**What is the implication of the main finding?**

- Vigilant monitoring for autonomic instability is critical in pediatric GBS.
- Early recognition and timely intervention can improve outcomes.

**Abstract:**

**Background:** Guillain-Barré syndrome (GBS) is an acute immune-mediated polyneuropathy often associated with autonomic dysfunction. Although transient cardiovascular instability is common, severe dysautonomia leading to cardiac arrest is rarely documented in children. **Methods:** We report the case of an 11-year-old previously healthy boy who initially presented with acute ophthalmoplegia and rapidly progressed to quadriplegia and areflexia. He developed fluctuating blood pressure and bradycardia, culminating in cardiac arrest due to asystole at 24 h after admission, requiring 17 min of resuscitation. **Results:** Electrophysiological studies confirmed a demyelinating polyneuropathy. Although intravenous immunoglobulin (IVIG) was initiated 5 h after admission, clinical improvement was achieved only after subsequent plasmapheresis on day 20, with the recovery of autonomic function by day 35. He was extubated on day 45 and discharged on day 83 with a near-complete recovery after prolonged intensive care and rehabilitation. **Conclusion:** This case highlights the potential for rapid and life-threatening autonomic instability in pediatric GBS. Unlike typical cases, the patient progressed to cardiac arrest within 24 h despite IVIG, highlighting the need to consider plasmapheresis for non-responders. Continuous hemodynamic monitoring is essential to prevent fatal outcomes, even in patients with initially mild or atypical presentations.

## 1. Introduction

Guillain-Barré syndrome (GBS) is an acute immune-mediated polyneuropathy that typically presents with ascending symmetrical weakness and areflexia. Since its initial characterization, several clinical subtypes have been identified [1,2]. Pediatric GBS differs from adult-onset cases in several clinically significant ways. Notably, children reach the disease nadir more rapidly—on average, in 6.3 days vs. 7.3 days in adults [3]. They also exhibit higher rates of bulbar involvement, such as swallowing or speech dysfunction (22.0% in children versus 14.8% in adults) [3]. Moreover, autonomic dysfunction is a far more prominent and worrisome feature in pediatric GBS [4]. Autonomic dysfunction occurs in up to 70% of cases and often manifests as transient symptoms such as blood pressure fluctuations or tachyarrhythmias [5,6,7]. Autonomic dysfunction often occurred 9–15 days after symptom onset, typically within 24–48 h of reaching maximum motor disability [2]. Among mechanically ventilated children, 39.1% exhibited autonomic dysfunction, compared to just 8.8% in adults, making autonomic dysfunction a strong and independent predictor for requiring mechanical ventilation in pediatric cases [3,4,5,6,7]. This suggests that children may develop autonomic instability more rapidly and severely, possibly due to immature compensatory mechanisms. Overall, pediatric GBS tends to progress faster, carries a greater morbidity linked to autonomic challenges, and shows more frequent cranial/bulbar involvement when compared to adult GBS.

However, severe dysautonomia leading to bradyarrhythmia, including cardiac arrest, is a rare but potentially fatal complication [5,6,7,8,9]. Few pediatric cases of GBS-associated cardiac arrest have been reported, and none have documented such rapid progression. This report describes the fastest known progression to cardiac arrest in a pediatric GBS patient.

## 2. Case Presentation

An 11-year-old previously healthy boy presented with acute right ptosis, dizziness, and vomiting. He denied any fever or upper respiratory symptoms within the past month and had neither TOCC (travel, occupation, contact, or cluster) exposure nor a recent vaccination history. He reported no specific sports-related activities and had no history of autonomic dysfunction. There was also no family history of cardiac or neurological disorders. The initial neurological examination was unremarkable except for diplopia. However, within twelve hours of admission, he developed a rapidly progressive ascending weakness that extended to the upper limbs, with muscle strength decreasing to 1/5 in all four extremities and areflexia. Concurrent tachycardia (101 bpm) and hypertension (158/75 mmHg) observed 4 h after admission raised concerns for autonomic dysfunction. The brain magnetic resonance imaging (MRI) performed at admission showed no evidence of brainstem involvement (Supplementary Figure S1). A cerebrospinal fluid analysis revealed albuminocytologic dissociation (a protein of 310 mg/dL and a white blood cell count of 9/mm^3^), raising suspicion for Guillain-Barré syndrome/Miller Fisher syndrome overlap. Intravenous immunoglobulin (IVIG) therapy was initiated 5 h after admission at a dose of 400 mg/kg/day for five consecutive days.

Twenty-four hours after admission, the patient developed episodes of bradycardia, diaphoresis, and fluctuating blood pressure, which culminated in cardiac arrest due to asystole. According to the family’s report, the patient was awake without a sedative drug and showed no signs of respiratory distress. Fingertip bedside monitoring revealed bradycardia (56 beats/min) with normal oxygen saturation prior to the event. However, no blood gas analyses or respiratory function measurements, such as forced vital capacity, were available before the arrest. A sudden onset of asystole was then witnessed by his mother. The return of spontaneous circulation (ROSC) was achieved after 17 min of resuscitation, during which the patient received intravenous epinephrine (1:10,000) at 0.1 mg/kg every 3 min for a total of five doses. He was intubated, placed on mechanical ventilation, and transferred to the pediatric intensive care unit (PICU).

**Figure 1:**
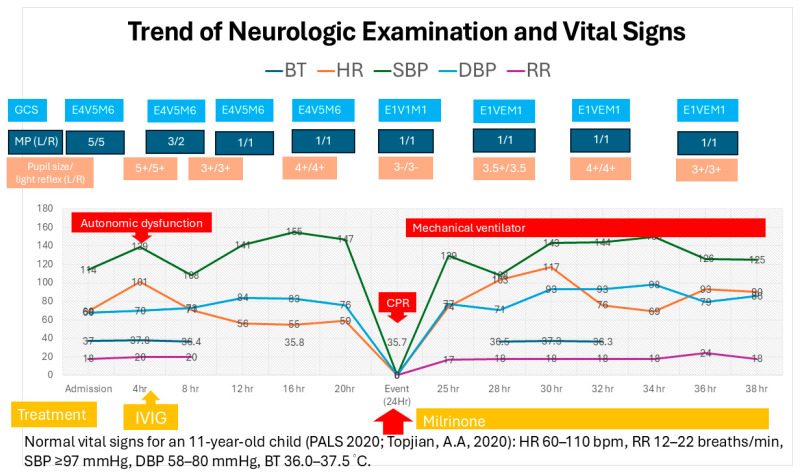
Progressive development of hypertension and bradycardia was noted within hours after admission, raising concerns for autonomic dysfunction preceding the event. Following the event, sustained hypertension and the emergence of tachyarrhythmias were documented over the subsequent 12 h. Abbreviations: GCS: Glasgow Coma Scale; MP: muscle power; L/R: left/right; BT: body temperature; HR: heart rate; SBP: systolic blood pressure; DBP: diastolic blood pressure; RR: respiratory rate; IVIG: intravenous immunoglobulin; and CPR: cardiopulmonary resuscitation. For reference, normal vital signs for an 11-year-old child (PALS 2020): HR 60–110 bpm, RR 12–22 breaths/min, SBP ≥97 mmHg, DBP 58–80 mmHg, and BT 36.0–37.5 °C (ref. [10]).

Electrophysiological studies confirmed severe demyelinating polyneuropathy with secondary axonal involvement. Serological testing for anti-ganglioside antibodies was negative. Blood pressure fluctuations and tachyarrhythmias were also observed (trend shown in Figure 1). By hospital day 10, no significant neurological improvement was observed, prompting the initiation of a five-cycle course of plasmapheresis. After completing 10 days of treatment, extraocular movement improved, and by day 20, the patient was able to follow commands and shake his head. Upper limb movement and autonomic function showed improvement by day 35. He was successfully extubated on hospital day 45. On day 54, he was transferred to the general pediatric ward for further physical rehabilitation. He was discharged on hospital day 83 with muscle strength graded 4/5 in all limbs. Table 1 summarizes the clinical examination findings and the responses to interventions.

## 3. Discussion

This case highlights the rapid progression and potentially life-threatening autonomic complications that can arise in pediatric Guillain-Barré syndrome (GBS) [8]. Notably, children reach the disease nadir more rapidly—on average, in 6.3 days [3]. Autonomic dysfunction often occurred 9–15 days after symptom onset, typically within 24–48 h of reaching maximum motor disability [4]. Although these symptoms are often transient, dysautonomia in children can be unpredictable and is frequently underrecognized. Bradyarrhythmias, including cardiac arrest, are uncommon but may occur suddenly and without warning [5,6,7,8,9]. While most pediatric GBS cases progress over days, this patient deteriorated within hours, akin to rare cases reported by Pfeiffer et al. [8].

Severe dysautonomia in Guillain-Barré syndrome (GBS) can occur even in the absence of detectable ganglioside antibodies, reflecting broader immune-mediated mechanisms. Antibodies against nodal or paranodal proteins (e.g., pan-neurofascin, contactin-1, and Caspr) and ganglionic acetylcholine receptor (gAChR) antibodies have been associated with fulminant disease and profound autonomic instability, often with a limited response to standard IVIG therapy [11,12,13,14]. Immune-mediated injuries to small autonomic fibers and the disruption of baroreflex pathways further exacerbate cardiovascular dysregulation [15].

It is essential to consider differential diagnoses of bradycardia based on the lesion’s localization. Evidence that tachycardia preceded cardiac arrest may indicate peripheral vascular insufficiency or autonomic shock. Alternatively, the presentation could reflect Cushing’s triad—hypertension, bradycardia, and irregular respiration—suggesting elevated intracranial pressure, possibly involving the fourth ventricle [16]. Another potential mechanism is primary failure of the efferent thoraco-cardiac sympathetic pathway, either at the stellate ganglion or along its preganglionic course. This failure could lead to unopposed vagal tone, resulting in sinus bradycardia or sinus arrest. Experimental and translational studies highlight the role of stellate ganglion and cardiac autonomic inputs in modulating conduction and repolarization, providing a mechanistic link to bradyarrhythmia and cardiac arrest [17,18]. Prolonged PR intervals or QT prolongation may further indicate the disruption of intrinsic cardiac innervation and autonomic balance [17,18]. If direct involvement of efferent sympathetic fibers is suspected, ancillary signs such as pupillary abnormalities could support the diagnosis; however, these signs may be difficult to assess in a sedated child.

Additionally, hypercapnic hypoventilation may have contributed to a downward spiral of cardiac insufficiency, culminating in arrest, suggesting that ventilatory compromise and autonomic dysfunction likely acted synergistically in the pathophysiological cascade [19]. In pediatric populations, autonomic dysfunction is well-recognized in Guillain-Barré syndrome and has been associated with severe bradycardia and even asystolic arrest, highlighting the importance of vigilant autonomic monitoring [5,6,7,8,9].

Diagnosis is based on clinical suspicion, supported by cerebrospinal fluid analysis showing albumin-cytologic dissociation, and confirmed by nerve conduction studies [2]. Although IVIG and plasmapheresis are generally considered equally effective first-line therapies, the delayed response to IVIG raises questions about its efficacy in fulminant dysautonomia [20]. Some patients—particularly those with axonal variants—respond poorly to IVIG but improve after early plasmapheresis [4,6]. In such rapidly progressive cases, the delayed escalation of therapy may increase the risk of catastrophic autonomic complications, including cardiac arrest. Early recognition of fulminant autonomic involvement, close hemodynamic monitoring, and the timely initiation of plasmapheresis should be considered, even when initial IVIG therapy has already been administered [4,6].

Given the potential for sudden autonomic deterioration, continuous cardiac monitoring is essential, especially during the early phase of the illness [2]. The timely recognition of autonomic instability and proactive management of bradyarrhythmias are critical to preventing catastrophic outcomes, including cardiac arrest [5,6,7,8,9]. This case highlights that even pediatric patients presenting with initially mild limb weakness may be at risk and warrant close and vigilant supportive care.

## 4. Conclusions

GBS-related dysautonomia can precipitate sudden cardiac arrest in children. Vigilant monitoring of autonomic function and timely intervention are essential to reducing morbidity and mortality. We recommend PICU admission for all pediatric GBS patients presenting with hypertension and bradycardia. Prophylactic temporary pacing should also be considered for pediatric GBS patients exhibiting early bradycardia.

## Figures and Tables

**Table 1:** Summary of examination findings and responses to interventions.

**Examination Findings**	
Examination	Finding
Physical examination	Right eye ptosis with progressive limb weakness
Brain MRI	No evidence of brainstem involvement
CSF analysis	Albuminocytologic dissociation (protein: 310 mg/dL; WBC: 9/mm^3^)
Electrophysiological studies (NCV)	Severe demyelinating polyneuropathy with secondary axonal involvement
Anti-ganglioside antibodies	Negative
**Intervention Responses**	
Intervention	Response
IVIG	No response
Plasmapheresis	Gradual improvement

MRI: magnetic resonance image; CSF: cerebrospinal fluid; WBC: white blood cell; NCV: nerve conduction velocity; and IVIG: intravenous immunoglobulin.

## Data Availability

No datasets were generated or analyzed during the current study, The data presented in this study are available on request from the corresponding author due to ethical reasons.

## Supplementary Materials

The following supporting information can be downloaded at: https://www.mdpi.com/article/10.3390/children12101379/s1, Figure S1: Brain magnetic resonance imaging (MRI).
