# Supplementary material for: Pediatric Cardiac Arrest Secondary to Guillain-Barré Syndrome-Induced Dysautonomia
Source: Children (Basel). 2025 Oct 13;12(10):1379. doi: 10.3390/children12101379 (PMC12564891; doi:10.3390/children12101379)
Supplement: Supplementary file 1 [file children-12-01379-s001.zip › children-3812630-supplementary.pdf]

Supplementary Figure S1: Brain magnetic resonance imaging (MRI)

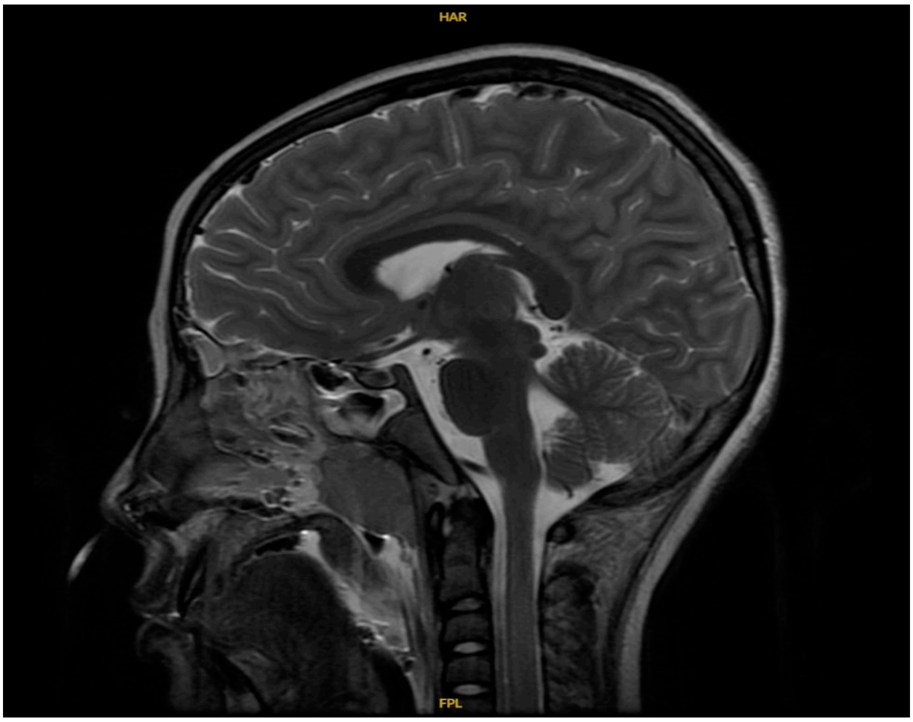

Brain magnetic resonance imaging (MRI) showed no evidence of brainstem involvement at admission.
